# When Is Simultaneous Recording Necessary? A Guide for Researchers Considering Combined EEG-fMRI

**DOI:** 10.3389/fnins.2021.636424

**Published:** 2021-06-29

**Authors:** Catriona L. Scrivener

**Affiliations:** MRC Cognition and Brain Sciences Unit, University of Cambridge, Cambridge, United Kingdom

**Keywords:** simultaneous EEG and fMRI, multimodal neuroimaging, EEG-informed fMRI, fMRI-informed EEG, combined recording

## Abstract

Electroencephalography (EEG) and functional magnetic resonance imaging (fMRI) provide non-invasive measures of brain activity at varying spatial and temporal scales, offering different views on brain function for both clinical and experimental applications. Simultaneous recording of these measures attempts to maximize the respective strengths of each method, while compensating for their weaknesses. However, combined recording is not necessary to address all research questions of interest, and experiments may have greater statistical power to detect effects by maximizing the signal-to-noise ratio in separate recording sessions. While several existing papers discuss the reasons for or against combined recording, this article aims to synthesize these arguments into a flow chart of questions that researchers can consider when deciding whether to record EEG and fMRI separately or simultaneously. Given the potential advantages of simultaneous EEG-fMRI, the aim is to provide an initial overview of the most important concepts and to direct readers to relevant literature that will aid them in this decision.

## Introduction

The ideal neuroimaging tool would provide a minimum of millimeter spatial resolution at millisecond temporal resolution, enabling researchers to obtain a detailed map of neural function in a living brain. Currently, this method does not exist, and we rely on the synthesis of information from a mixture of methods, each with their own strengths and weaknesses. Among the most common methods available are electroencephalography (EEG), and functional magnetic resonance imaging (fMRI), providing non-invasive measures of brain activity at different spatial and temporal scales. This article focuses on the increasingly common method of combining EEG and fMRI signals by recording them simultaneously.

The concurrent acquisition of EEG and fMRI has the ambitious aim of improving the spatial and temporal limitations of respective measures, promising increased understanding of brain function. Some of the first applications of simultaneous EEG-fMRI aimed to improve localization of epileptic seizures in epilepsy patients, where increased spiking in the EEG can be correlated with activation in contributing brain areas (Ives et al., 1993). In addition, epileptic spikes recorded in the EEG can be used to indicate the onset of epileptic events and inform the time course of fMRI analysis (Bénar et al., 2003, 2006; Vulliemoz et al., 2009; Gotman and Pittau, 2011). In a similar application, EEG signals can be used to indicate sleep phases and facilitate partitioning of simultaneously recorded fMRI signal (Portas et al., 2000; Horovitz et al., 2008).

The primary advantage of simultaneous EEG-fMRI recording is that it allows one to obtain two complementary data sets capturing identical brain activity. There are many circumstances in which separate recordings of EEG and fMRI would be unlikely to contain the same information, for example in resting state or decision-making paradigms (Mayhew et al., 2013; Pisauro et al., 2017). During combined recording, the same neural activity contributes to the EEG and fMRI data at each trial. This assumption is necessary for many existing EEG-fMRI analysis methods, such as EEG-informed fMRI or resting state network analysis (Eichele et al., 2005; Mantini et al., 2007; Wirsich et al., 2021) and provides a rich data set for cognitive and clinical investigation. Other differences between separate EEG and fMRI experiments are also avoided by a single recording session, such as variance in sensory stimulation, stimuli habituation, subject position, and preparation time; all of which may have subtle but important impacts on the recorded data (Herrmann and Debener, 2008).

The primary disadvantage of simultaneous EEG-fMRI recording is that each data set is negatively impacted by the presence of the other. EEG data recorded inside the MRI environment contains gradient (Mandelkow et al., 2006), ballistocardiogram (BCG; Debener et al., 2008; Marino et al., 2018a), pump (Rothlübbers et al., 2015), and ventilator related artifacts (Nierhaus et al., 2013). The EEG data quality is therefore reduced compared to a separate recording session in a shielded environment, which affords greater flexibility regarding the type of EEG cap used (for a comparison see Mathewson et al., 2017). For example, auditory event-related potentials (ERPs) can be influence by the increased noise inside the MRI scanner (Mulert et al., 2004), and there is some evidence of changes to cognitive ERPs and steady-state visually evoked potentials (Novitski et al., 2001, 2003; Sammer et al., 2005) when they are recorded during EEG-fMRI. However, other studies report comparable ERP results inside and outside the MRI environment, and this may depend on factors such as the ERP of interest and the signal-to-noise ratio of the recorded data (Becker et al., 2005; Comi et al., 2005; Bregadze and Lavric, 2006; Mayhew et al., 2010). The MRI signal can also be impacted by combined recording, as the EEG electrodes increase inhomogeneity in the magnetic field and reduce MRI signal (Mullinger et al., 2008c). However, the continued development of novel methods to remove artifacts and improve data quality in simultaneously recorded data provides optimism for the future of EEG-fMRI (Marino et al., 2018b; Bullock et al., 2021) and continues to reduce the weight of this disadvantage.

**FIGURE 1:**
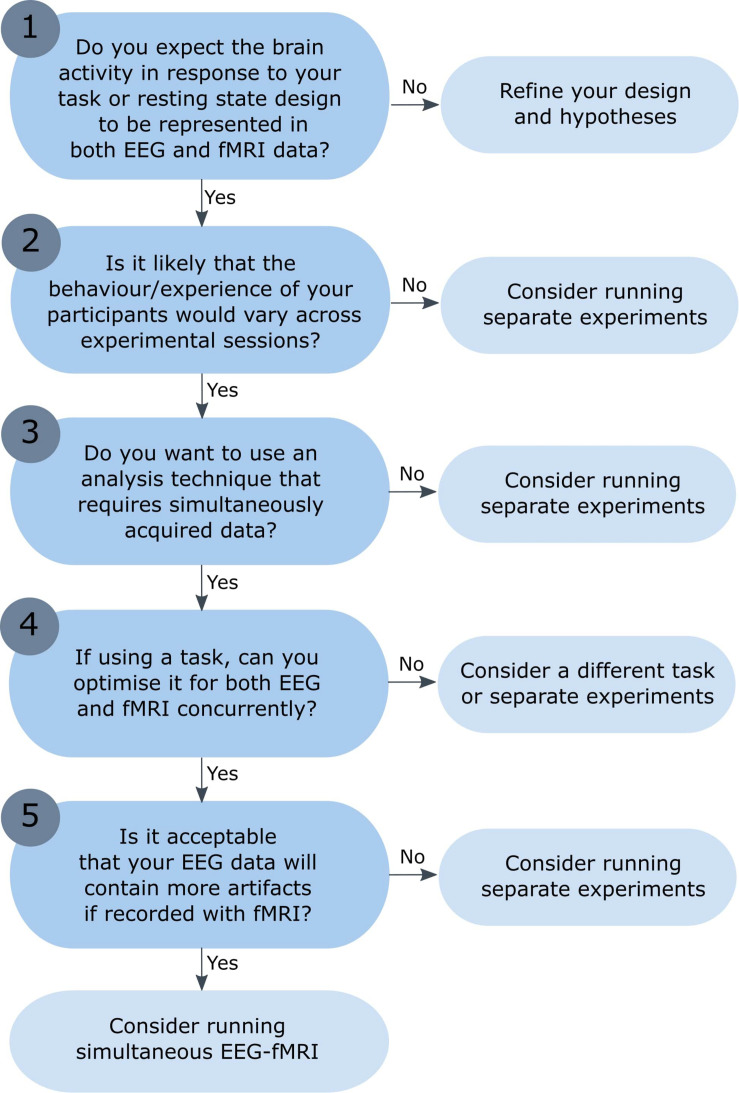
Flow chart: is simultaneous EEG-fMRI necessary? The numbered questions correspond to numbered sections of the text, which describe each question and the possible outcomes in more detail. While the experimental process will not necessarily take the linear progression presented here, the aim is to offer a set of key questions to answer before undertaking simultaneous EEG-fMRI recording. The reader is directed to the critical literature referenced throughout each section to aid them in their response to each question.

Simultaneous EEG-fMRI is increasingly used to investigate brain activation in healthy subjects and a range of methods have been proposed for data integration (for other more detailed conceptual and methodological reviews, see Ritter and Villringer, 2006; Huster et al., 2012; Laufs, 2012; Jorge et al., 2014). However, combined EEG-fMRI is not necessarily better than separate sessions, and researchers should consider their research question and experimental design before recording EEG and fMRI simultaneously. This article highlights the challenges faced when recording simultaneous EEG-fMRI, including the nature of the signals that we record and when we can expect them to overlap. Presented here is a flow chart to help researchers decide whether simultaneous EEG-fMRI is necessary, or whether separate EEG and fMRI experiments would be more appropriate (see Figure 1). The flow chart begins with the assumption that researchers would like to acquire both fMRI data and EEG data, but are deciding whether these need to be recorded during the same experimental session, or separately (the decision whether to record just EEG *or* just fMRI data would require an additional set of statements). Although this list of questions is not exhaustive, and the experimental process will not always take the linear progression presented here, this article points to useful resources and aims to provide a good starting point for any researcher considering combined EEG-fMRI recording.

## 1. Do You Expect the Brain Activity in Response to Your Task or Resting State Design to Be Represented in Both EEG and fMRI Data?

The first question is perhaps the most important, but also the most difficult to answer. A pre-requisite for combined EEG-fMRI is that you expect both modalities to capture and reflect the activation that you hypothesize to find. If you do not expect this, then there is little to gain from recording both data sets, either simultaneously or not. If this is the case, you should refine your experimental design and hypotheses before coming back to this question. An ideal combined EEG-fMRI recording would provide a data set with an integrated view of the underlying neural activity, such that the EEG, fMRI, and behavioral measures have some degree of overlap. You could consider this using a Venn diagram with three overlapping circles, where the center portion captures information from all three measures (Jorge et al., 2014). However, there are multiple reasons why this may not always be the case, and it is possible for correlations to occur between behavior and each modality separately, without any overlap between the EEG and fMRI signals themselves. [For an in-depth discussion on the neural sources of both modalities, see the chapters on the physiological basis of EEG and blood oxygen level dependent (BOLD) signals in Ullsperger and Debener, 2010, and the review on EEG-fMRI integration from Rosa et al., 2010].

As EEG and fMRI are sensitive to activity at different spatial and temporal scales, it is plausible to find a decoupling between electrophysiological and hemodynamic signals (Nunez and Silberstein, 2000; Rosa et al., 2010; Jorge et al., 2014). For example, fMRI BOLD can be recorded in the absence of EEG if the neural activity is not detectable at the scalp. This can occur if the electrophysiological activity is non-synchronized, forms a closed source, or is present in deep sources that are subject to volume conduction and signal decay (Henze et al., 2000; but see Seeber et al., 2019). It is also possible for areas with high metabolic load to contribute to BOLD but not to EEG (Ritter and Villringer, 2006).

Similarly, it is possible to detect EEG in the absence of BOLD if the sources of EEG signals do not consume enough energy to facilitate the hemodynamic changes detectable in fMRI (Mulert and Lemieux, 2009). Dynamics with high temporal resolution may also be missed or smoothed by the sampling rate of fMRI BOLD. Further, reduced inhibitory cell activity can decrease metabolic load but increase pyramidal activity (Nunez and Silberstein, 2000), resulting in divergent effects in EEG and fMRI.

Given these possibilities, it is beneficial to use a task/experiment that has already been run separately for EEG and fMRI with replicable results. If previous research has found measurable signals in both data sets using a similar paradigm, then you may not need to run these experiments again. However, if you have designed a new paradigm, or plan to use a new analysis method, it may be beneficial to check that your expected signal can be found in both EEG and fMRI before running combined recording. A further point to emphasize here is that researchers should have some idea of what they expect to find in both modalities using their analysis technique(s). When confronted with a large data set from simultaneous recording, it is important to have some idea of the analysis pipelines that will be run and what results it is reasonable to expect. The flexibility and extent of possible analysis pipelines can result in different conclusions, even from the same data set, in the hands of different researchers (Botvinik-Nezer et al., 2020). With more data comes an increased chance of spurious results, and without a clear direction for analysis it is possible to find *something*, even if not meaningful. Registered reports and pre-registered analysis pipelines go some way to ensure the reproducibility of neuroscience research, by illuminating planned versus *post hoc* hypotheses and analysis (Gorgolewski and Poldrack, 2016).

In summary, researchers should be confident that their signal of interest is detectable with both EEG and fMRI, from previous research or knowledge of the neural source. If this is not the case, then simultaneous EEG-fMRI may not provide any additional information, and relationships between the EEG and fMRI signals may not be detectable.

## 2. Is It Likely That the Behavior/Experience of Your Participants Would Vary Across Experimental Sessions?

Question 2 asks the researcher if it is likely that the behavior/experience of their participants would vary across experimental sessions, for example in a learning or emotional task. If this is the case, then even if identical paradigms were recorded using the same participants across separate EEG and fMRI experiments, it would be difficult to ensure that the brain activity at each individual trial was the same. Another example is the application of simultaneous EEG-fMRI recording to investigate resting state networks, given that spontaneous activation across sessions cannot be matched (Mantini et al., 2007; Scheeringa et al., 2008; Britz et al., 2010; Marino et al., 2019; Wirsich et al., 2021). If it is likely that the behavior/experience of your participants would vary across experimental sessions, then simultaneous recording is beneficial. Other differences between separate EEG and fMRI experiments are avoided by implementing a single recording session, such as variance in sensory stimulation, stimuli habituation, subject position, and preparation time; all of which may have subtle but important impacts on the recorded data (Herrmann and Debener, 2008). However, if stability across sessions is likely, then separate recordings may be sufficient.

## 3. Do You Want to Use an Analysis Technique That Requires Simultaneously Acquired Data?

Question 3 asks the researcher if their analysis requires data recorded during the same session. The answer to this question is dependent on several factors, including the answer to question 2 above, and the chosen method of analysis. If you are certain that the brain activation in response to two separate sessions will be relatively stable, and you can classify each trial type predictably (for example, in some well controlled visual stimulation experiments), then you may be able to run your analysis on separately recorded data. However, if you predict that the activity at each trial will vary across experimental sessions, then simultaneous recording may be necessary for most analysis techniques (for example, if the response of the participant determines the trial type/condition).

The existing analysis methods for combined EEG-fMRI can be broadly grouped into two categories; symmetrical and asymmetrical analysis (for more detailed reviews, see Huster et al., 2012; Jorge et al., 2014). In asymmetric analysis, one modality is used as a predictor for the other. EEG-informed fMRI uses values extracted from single-trial EEG as predictors in a standard fMRI GLM analysis (general linear model). This identifies voxels with activation that co-varies with fluctuations in the EEG signal over time. Example EEG features include ERPs and frequency-band power fluctuations, the choice of which will determine the conclusions that can be drawn from the results (Laufs et al., 2003; Novitskiy et al., 2011).

An ongoing challenge for the application of EEG-informed fMRI is to maximize the signal-to-noise ratio in single trial estimates of EEG activity. Methods to improve the efficiency of data pre-processing before integration are therefore highly important to the field of EEG-fMRI and continue to be developed. Key examples include linear classifiers (Goldman et al., 2009; Walz et al., 2015), autoregressive models (Nguyen et al., 2014), spatial Laplacian filters (Liu et al., 2016), and functional source separation (Porcaro et al., 2010). Unless it can be assumed that single-trial values will be stable across two experimental sessions, and not negatively influenced by habituation or learning effects, ERP-informed fMRI method will generally require simultaneously recorded data (Debener et al., 2006; Bagshaw and Warbrick, 2007). Another important consideration for single trial EEG analysis is that it relies on EEG effects that are present at the within-subject level. Not all ERPs are stable across participants if analyzed at a single-subject level (e.g., Petit et al., 2020), which may negatively impact single trial ERP-informed fMRI analysis. Therefore, it may be beneficial to check the variance across subjects and sessions of an EEG measure before using in combined EEG-fMRI analysis.

In the opposite direction, fMRI-informed EEG uses fMRI GLM results to guide and/or constrain EEG source reconstruction, which benefits from the additional spatial information provided by fMRI. The strength of the assumed overlap between EEG sources and fMRI BOLD will determine the source reconstruction constraints (see the discussion in Chapter 3.7 of Ullsperger and Debener, 2010, for a mathematical overview). Arguably, this could also be achieved using separately recorded data, but this decision will be influenced by the same considerations mentioned above for EEG-informed fMRI.

In symmetrical analysis, researchers avoid giving preference to one modality by modeling relationships between the data or calculating joint independent components (Daunizeau et al., 2007; Moosmann et al., 2008). Current symmetrical analysis can be divided into data-driven and model-based methods. Independent component analysis (ICA; Calhoun et al., 2006; Lei et al., 2011) and methods based on information theory (Ostwald et al., 2011) are data-driven, as they do not require modeling of hemodynamics or neurovascular coupling. In contrast, dynamic causal modeling (Friston et al., 2003; Nguyen et al., 2014) and other model-based methods attempt to determine the underlying neural components of EEG and fMRI using individual forward models (Rosa et al., 2010).

There are several ICA based methods for EEG-fMRI analysis, including parallel ICA and joint ICA. In the parallel application, temporal ICA is typically run on the EEG data, whereas spatial ICA is run on the fMRI data (Liu and Calhoun, 2007; Eichele et al., 2008). Components are then matched, for example, by correlating the component time series. As the temporal sequence of events is preserved, this method also assumes consistent activation across modalities in single trials, and may require simultaneous recording. In joint ICA, EEG and fMRI data is included in the same ICA decomposition, such that the mixing matrix contains information from both modalities (Moosmann et al., 2008; Calhoun et al., 2009). It is possible to conduct joint ICA with averaged ERPs and fMRI contrast maps, or with single trial data, of which the former can feasibly be run on separately recorded data (Calhoun et al., 2006).

Although many of the analysis methods mentioned above ideally require simultaneous recording, methods designed for combining magnetoencephalography (MEG) and fMRI could also be applied to separately recorded EEG and fMRI data. M/EEG fMRI fusion methods based on representation similarity analysis (RSA) search for shared information across data sets condition-by-condition, rather than trial-by-trial, and therefore do not require simultaneous recording (Cichy and Oliva, 2020). These multivariate methods have additional advantages; they identify differences in the patterns of activation across regions or time points, rather than the overall activation as identified in univariate analysis. Additionally, all M/EEG channels are typically entered into the analysis, which may be more informative than the few channels that are usually selected for EEG-informed fMRI analysis as a larger proportion of the available data is used.

In summary, if you plan to run traditional EEG and fMRI analyses separately, using averages over trials or representations of shared information, then separate recording may be sufficient. However, if you plan to run combined EEG-fMRI analysis with the assumption that the same behavior and neurological activity is represented in both modalities at each trial, then concurrent recording is beneficial.

## 4. If Using a Task, Can You Optimize It for Both EEG and fMRI Concurrently?

Question 4 is important for the design of a combined EEG-fMRI experiment, and asks if the task can be optimized for both EEG and fMRI. Traditional EEG experiments are often fast with short trial durations, given that researchers are usually interested in activity within the first 600 ms after stimulus onset. The BOLD response measured using fMRI is much slower, and therefore fast paradigms must be designed with caution. Researchers may consider adding larger inter-trial intervals and jitter between image presentations to ensure that the BOLD in response to separate trials can be separated (Amaro and Barker, 2006). Furthermore, not all fMRI safe EEG equipment can be used in conjunction with all fMRI acquisition techniques such as multiband sequences, therefore constraining the fMRI sequence design (Chen et al., 2020). An advantage of separate recording sessions is that the task and recording setup can be individually optimized for each modality, and therefore the signal-to-noise maximized in both data sets.

## 5. Is It Acceptable That Your EEG Data Will Contain More Artifacts Have a Reduced Signal-to-Noise Ratio When Recorded Inside the MRI Scanner?

The final question asks researchers if they are happy to accept that their EEG data will contain more artifacts and have a reduced signal-to-noise ratio when recorded inside the MRI scanner. Unfortunately, it is not possible for anyone to accurately quantify the loss of signal that will occur for a given paradigm or EEG feature. However, the EEG data recorded in the MRI environment will have additional artifacts that need to be removed (Ritter and Villringer, 2006; Marino et al., 2019; Bullock et al., 2021). Strategies for the removal of these artifacts have variable success, and it is possible that additional data sets will need to be excluded due to pre-processing failures (e.g., Scrivener et al., 2020).

When EEG is recorded during MRI acquisition, several additional EEG artifacts are incurred. The first is caused by the gradient pulse, and therefore known as the gradient artifact. As this is related to the sequence of the MRI scanner, which is known, and is stable over time, the gradient artifact can be removed by subtracting a template of its form (see Allen et al., 2000). This is facilitated by synchronizing the EEG and fMRI clocks, which improves removal of the gradient artifact (Mandelkow et al., 2006; Mullinger et al., 2008a).

The second artifact is the BCG, which is considerably harder to remove, and a reliable solution to this is yet to be achieved. The BCG artifact is related to the heartbeat of the participant lying down in the scanner. More specifically, expansion and contraction of arteries in the scalp cause movements in the electrodes and wires in the EEG cap (Goldman et al., 2000; Debener et al., 2008). This movement of blood also influences the static magnetic field and can result in artifacts with larger power than the EEG signal of interest (Ritter and Villringer, 2006). With similarities to the removal of the gradient artifact, one method used to remove the BCG is to construct a template of the heartbeat artifact, identify its occurrence across the recording, and subtract it from the EEG signal (Allen et al., 1998).

However, there are several factors that reduce the success of this method. Unlike the gradient artifact, the heartbeat of the participant is not stable over time, which can make its removal more of a challenge. Several other methods for removing the BCG artifact have been suggested, for example, ICA (Srivastava et al., 2005), adaptive filtering based on a time varying finite impulse response (Bonmassar et al., 2001), and adaptive optimal basis set methods (Marino et al., 2018b). However, no method claims to successfully remove all BCG artifacts for all participants, and they are not consistently applied across studies (for a review of existing methods, see Abreu et al., 2018 and Bullock et al., 2021).

The third artifact present in EEG recorded inside the MRI environment is caused by the helium pump, which results in widespread peaks across the frequency spectrum, far above the amplitude range of normal EEG (Mullinger et al., 2008b). Given the spread across frequencies, and the difficulty in distinguishing true neural signal from helium pump noise, this can be difficult to remove (but see Rothlübbers et al., 2015). A further complication is the large between-site differences in helium artifact, driven by factors such as the scanner manufacturer and physical set up (Neuner et al., 2014). One way to avoid this artifact is to switch off the helium pump before running the experiment (Laufs et al., 2008). However, as the helium pump is essential for the continued functioning of the MRI scanner, this cannot be left switched off for long time periods.

The MRI signal can also be affected by combined recording, with greater impact reported at higher field strengths (Mullinger et al., 2008c; Jorge et al., 2015). The EEG electrodes increase inhomogeneity in the magnetic field and reduce MRI signal (Mullinger et al., 2008c; Abreu et al., 2018), as well as producing artifacts at the location of EEG electrodes (de Munck et al., 2012; Scrivener and Reader, 2021). However, as the distortion and signal drop-out caused by electrodes is located at the scalp, the signal within the brain is not significantly affected (Mullinger et al., 2008c). Further, several studies have reported comparable BOLD sensitivity with and without the presence of an EEG cap (Luo and Glover, 2012; Klein et al., 2015).

Some researchers have also reported spurious correlations between EEG and fMRI signals that are related to motion artifacts, rather than a common neural source. For example, EEG power in the frequency domain was found to be significantly higher during trials with high motion, compared to low motion, especially in low frequency bands (Fellner et al., 2016). The presence of additional EEG artifacts may also influence ICA based analysis, as a larger proportion of the ICA components will relate to artifacts than in a standard EEG experiment. Given that the number of identified ICA components is restricted to the maximum number of recording channels, this may reduce the ability to separate meaningful EEG components (Ullsperger and Debener, 2010).

## Future Directions

Recent technological and methodological developments continue to expand and improve the application of combined EEG-fMRI. For example, real-time pre-processing and feedback of sensorimotor EEG activation has been implemented during EEG-fMRI recording, enhancing task related activity during motor imagery (Zich et al., 2015). It is therefore possible to conduct online EEG analysis and use the outcome measure to influence participant behavior and subsequent neural activation. The complex concurrent application of TMS-EEG-fMRI has also been implemented, suggesting that strong pre-stimulus alpha power is associated with a reduced TMS-induced BOLD response in the motor execution network (Peters et al., 2020). An interesting extension of this work would be to use online EEG measures, such as alpha peak or trough onsets, to inform the timing of TMS pulses delivered to the brain (Thut et al., 2011a,b), enabling a comparison of the TMS-induced changes to BOLD and behavior.

While most combined EEG-fMRI recordings are done at magnetic field strengths of 3T, several groups are developing methods to combine EEG with ultra-high field MRI imaging at 7T (Meyer et al., 2020; Philiastides et al., 2021). Despite the increased number of artifacts associated with combined EEG-fMRI recording over 3T (Mullinger et al., 2008c; Neuner et al., 2013; Jorge et al., 2015), ultra-high field MRI measures provide increased spatial resolution at the mesoscopic level (Balchandani and Naidich, 2015). Combining this with EEG can therefore facilitate precise mapping between EEG signals and BOLD activation across individual layers of the cortex (e.g., Scheeringa et al., 2016), with greater detail than can be achieved at lower field strengths.

## Summary

The aim of this article is to provide a resource for researchers considering whether to record EEG and fMRI separately or simultaneously. Presented here are a series of questions for the researcher to consider in reaching a decision. In summary, separate recordings are sufficient if; (a) you plan to run traditional EEG and fMRI analyses separately, using averages over trials, rather than combined analysis and single-trial data; (b) you assume that participant behavior and neurological responses would be relatively stable across experimental sessions; and (c) if you cannot find a suitable paradigm that can be optimized for EEG and fMRI concurrently. If you do not know what to expect in the individual modalities or how you would analyze your data, it may be beneficial to run pilot studies in each modality first before coming back to the question of simultaneous EEG-fMRI. There are several advantages to separate recording, including the ability to optimize the recording parameters for each modality separately, without requiring specialized fMRI safe EEG equipment that may constrain the available fMRI sequences. The data quality of separate data sets will also be greater, and the likelihood that subjects need to be excluded from analysis is reduced by the smaller number of possible artifacts.

However, simultaneous EEG-fMRI may be justified if; (a) you can reasonably expect your signal of interest to be detected in both EEG and fMRI signals; (b) you know what to expect from each modality individually and are therefore interested in running combined analysis to extract simultaneous temporal and spatial information; (c) you plan to run combined EEG-fMRI analysis with the assumption that the same behavior and neurological activity is represented in both modalities at each trial; and (d) you expect that this behavior and neurological activity would vary across experimental sessions or you are measuring spontaneous activity during resting state. An advantage of simultaneous recording is the mitigation of potential confounds associated with multiple testing sessions, such as habituation, learning, or differences in set up and participant experience.

Overall, the aim of this article is to equip new researchers with the resources needed to make an informed decision regarding the necessity of simultaneous EEG-fMRI. As multi-modal neuroimaging requires additional time, equipment, and financial resources, it is important to thoroughly consider the recording options available. Ongoing technological and methodological developments continue to facilitate the successful application of combined EEG-fMRI to ask questions about the brain and behavior with increasing precision, and it will no doubt continue to be a powerful tool in cognitive neuroscience.

## Author Contributions

The author confirms being the sole contributor of this work and has approved it for publication.

## Conflict of Interest

The author declares that the research was conducted in the absence of any commercial or financial relationships that could be construed as a potential conflict of interest.
